# Enhancing Erucic Acid and Wax Ester Production in *Brassica carinata* through Metabolic Engineering for Industrial Applications

**DOI:** 10.3390/ijms25126322

**Published:** 2024-06-07

**Authors:** Misteru Tesfaye, Eu Sheng Wang, Tileye Feyissa, Cornelia Herrfurth, Teklehaimanot Haileselassie, Selvaraju Kanagarajan, Ivo Feussner, Li-Hua Zhu

**Affiliations:** 1 Department of Plant Breeding, Swedish University of Agricultural Sciences, P.O. Box 190, SE-234 22 Lomma, Sweden; misteru.tesfaye.woldeyohannes@slu.se (M.T.); eu.sheng.wang@slu.se (E.S.W.); selvaraju.kanagarajan@slu.se (S.K.); 2 Institute of Biotechnology, Addis Ababa University, Addis Ababa P.O. Box. 1176, Ethiopia; tileye.feyissa@aau.edu.et (T.F.); teklehaimanot.haileselassie@aau.edu.et (T.H.); 3 Department of Plant Biochemistry, Albrecht Haller Institute for Plant Science, University of Goettingen, Justus-von-Liebig-Weg 11, 37077 Goettingen, Germany; cornelia.herrfurth@biologie.uni-goettingen.de (C.H.); ifeussn@uni-goettingen.de (I.F.); 4 Service Unit for Metabolomics and Lipidomics, Goettingen Center for Molecular Biosciences (GZMB), University of Goettingen, Justus-von-Liebig Weg 11, 37077 Goettingen, Germany; 5 Department of Plant Biochemistry, Goettingen Center for Molecular Biosciences (GZMB), University of Goettingen, Justus-von-Liebig Weg 11, 37077 Goettingen, Germany

**Keywords:** *Brassica carinata*, erucic acid, industrial application, metabolic engineering and wax ester

## Abstract

Metabolic engineering enables oilseed crops to be more competitive by having more attractive properties for oleochemical industrial applications. The aim of this study was to increase the erucic acid level and to produce wax ester (WE) in seed oil by genetic transformation to enhance the industrial applications of *B. carinata*. Six transgenic lines for high erucic acid and fifteen transgenic lines for wax esters were obtained. The integration of the target genes for high erucic acid (*BnFAE1* and *LdPLAAT*) and for WEs (*ScWS* and *ScFAR*) in the genome of *B. carinata* cv. ‘Derash’ was confirmed by PCR analysis. The qRT-PCR results showed overexpression of *BnFAE1* and *LdPLAAT* and downregulation of RNAi-*BcFAD2* in the seeds of the transgenic lines. The fatty acid profile and WE content and profile in the seed oil of the transgenic lines and wild type grown in biotron were analyzed using gas chromatography and nanoelectrospray coupled with tandem mass spectrometry. A significant increase in erucic acid was observed in some transgenic lines ranging from 19% to 29% in relation to the wild type, with a level of erucic acid reaching up to 52.7%. Likewise, the transgenic lines harboring *ScFAR* and *ScWS* genes produced up to 25% WE content, and the most abundant WE species were 22:1/20:1 and 22:1/22:1. This study demonstrated that metabolic engineering is an effective biotechnological approach for developing *B. carinata* into an industrial crop.

## 1. Introduction

A rapidly growing global population and the decline in petroleum sources demand a search for renewable energy sources to reduce our dependence on fossil fuels. Vegetable oils and their derivatives possess a wide range of chemical and physical properties that could be tailored for various industrial applications, including as renewable bioenergy alternatives to fossil fuels [1,2].

*Brassica carinata* (carinata), also called Ethiopian mustard, is an amphidiploid (BBCC, 2*n* = 34) and evolved through natural hybridization between the diploid species *B. nigra* (BB, 2*n* = 16) and *B. oleracea* (CC, 2*n* = 18). The cultivation of carinata as an oil crop is largely limited to Ethiopia, while it is often grown as a leafy vegetable in other countries in Eastern and Southern Africa [3]. Due to its popularity as an industrial crop platform, carinata has spread to other countries such as Canada, India, Australia, Spain and the United States [4].

Carinata has become one of the potential oil crops for bio-industrial applications due to its naturally high levels of the very long-chain fatty acid, erucic acid (C22:1) (EA) [5]. EA and its derivatives are used in various industrial applications such as lubricants, detergents and film processing agents, as well as in cosmetics and pharmaceuticals [6,7,8]. In addition, oilseed brassicas with high EA content have a high iodine value and are thus more suitable for biodiesel or biofuel production [9]. EA is a very long-chain monounsaturated fatty acid (*cis-*13-docosenoic acid) with 22 carbon atoms and a double bond at the *cis*-13 position of the carbon chain. The biosynthesis of EA takes place in the cytosolic leaflet of the ER through a chain elongation reaction using oleic acid (C18:1) and a specific β-ketoacyl-CoA synthase (KCS) [10] as the initial substrate and catalytic enzyme, respectively [11]. The oil from carinata seeds is generally considered to have a high EA proportion, ranging from 31 to 46% in most natural germplasms and cultivars grown in Ethiopia [12,13,14]. The current market, however, demands high EA proportions beyond 46%, as demonstrated by high EA rapeseed cultivars grown in Europe and North America, which have 48% to 50% of EA in the seed oil [15,16]. The issue of food versus fuel debate is more prominent for rapeseed than carinata due to its large world production area coverage and as a source of edible oil. Thus, it is a better option to use carinata as feedstock for non-food industrial applications. Nowadays, carinata is considered a low-carbon-intensity feedstock that can be used in the production of drop-in renewable fuel or jet biofuel [17].

Owing to its naturally relatively high level of EA, carinata is also an attractive oilseed crop for producing wax esters (WEs). WEs are a class of neutral lipids with various industrial applications. WEs are widely used to produce surface coatings, printing inks and polishes, as well as for cosmetics and pharmaceutical applications. Although WEs are currently produced on a large scale from fossil reserves using chemical methods, such production cannot meet the increasing demand and is not environmentally friendly [18]. In this context, plants would be an attractive source for sustainable and environmentally friendly production of WEs. However, plant seeds do not normally produce WEs but mainly triacylglycerols (TAGs), but one exception is jojoba (*Simmodsia chinensis*), a desert plant that contains WEs in a large proportion as compared to TAGs in its seeds [19]. Jojoba, however, is less productive and is limited to growing in only hot and dry areas, and its oil is very expensive and usually used in the cosmetic industry [20].

A WE molecule is composed of a fatty acid esterified to a fatty alcohol. The biosynthesis of WEs is accomplished with the help of two enzymes, fatty acyl-coenzyme A (CoA) reductase (FAR), which converts fatty acyl-CoAs to fatty alcohols, and wax synthase (WS), which esterifies fatty acyl-CoAs to fatty alcohols to form WEs [21,22].

As a novel and modern breeding technology, genetic engineering has provided new possibilities for genetic studies and trait improvement in major crops. In carinata, the technique has been introduced [23,24,25,26], but the extent has been very limited compared to other major oilseed crops, such as rapeseed. Initially, the research was mainly focused on studies for developing efficient transformation methods for carinata, while the trait of focus has been modifying the fatty acid profile for developing high EA lines, with some promising results achieved. It has been reported that co-suppression and antisense repression of the *FAD2* gene increased the EA level by 12–27% and 5–19%, respectively [27], while downregulation of *FAD2* along with overexpression of crambe (*Crambe abyssinica*) *CaFAE* resulted in an increase in EA content by 16% in carinata [28]. The high EA results achieved so far, however, are not to the extent needed to satisfy the great demand for high EA products on the market. Additional efforts are needed to modify more key genes controlling the EA content. Earlier studies have indicated that the endogenous lysophosphatidic acid acyl-transferase (LPAAT) in *Brassica* spp. is unable to incorporate EA in the *sn-*2 position of the TAG backbone and thus limits the final EA content [29]. As demonstrated by Nath et al. [30], co-expression of *LdLPAAT* from *Limnanthes douglasii* and fatty acid elongase (*BnFAE1*) from *B. napus* were found to be effective in integrating EA into the *sn*-2 position of TAG and thus increasing EA content in rapeseed. Combining the simultaneous expression of the *LdLPAAT* and *BnFAE1* genes along with the downregulation of the *FAD2* gene, which competes for C18:1 with the *FAE1* gene, would further increase the EA content. This approach has been shown to be effective in crambe for obtaining stable high EA lines in subsequent generations [31,32]. In this study, we used the same transformation construct for increasing the EA content in carinata.

Metabolic engineering of plants for WE production was first demonstrated in Arabidopsis through the introduction of the *ScFAR*, *ScWS* and *ScFAE1* genes from jojoba [33], followed by major achievements in crambe (*Crambe abyssinica*), and it was also successfully tested in Camelina (*Camelina sativa*), carinata (*B. carinata*) and Lepidium (*Lepidium campestre*) [20,32,34]. So far, only one study has been reported with limited results on WE production in carinata [20]. More investigations are needed to provide strong evidence and support for using this approach to produce WEs in the species for industrial applications with broader perspectives.

In this study, we aimed to further demonstrate the potential of metabolic engineering of oil quality in carinata through *Agrobacterium*-mediated transformation for high EA content and WE production.

## 2. Results

### 2.1. Kanamycin Resistance

The results of the kanamycin resistance test showed that the shoot regeneration frequency started to decrease significantly when the kanamycin level reached 25 mg/L and above, and the regeneration percentage decreased to 4.7% when kanamycin was at 50 mg/L and was close to zero after 4 weeks of culture (Table 1). The calli that grew on the medium with 15 mg/L kanamycin were observed as yellowish, while the calli that grew at high kanamycin concentrations (75 and 100 mg/L) were found to be whitish-yellow. Accordingly, we have chosen 25 mg/L and 35 mg/L of kanamycin concentrations for the subsequent transformations in order to maintain a reasonable regeneration rate while reducing the frequency of escapes.

### 2.2. Confirmation of Transgenic Lines

**Figure 1:**
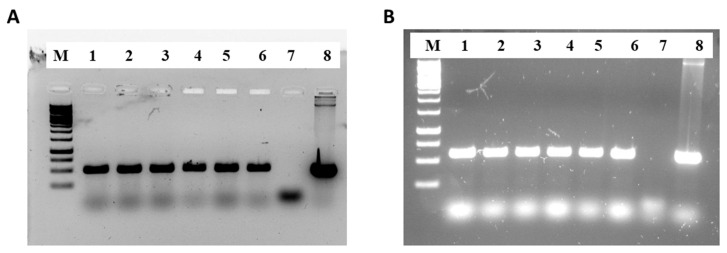
Integration of *BnFAE1* (**A**) and *LdLPAAT* (**B**) in the transgenic lines, shown by clear bands, while no bands are visible in the wild type. M: 1 kb DNA ladder from GeneRuler, L1–L6: transgenic lines, L7: wild type, L8: plasmid DNA.

Six transgenic lines were confirmed by PCR analysis, showing the integration of the *BnFAE1* (A) and *LdPLAAT* (B) genes, while no bands were observed in the wild type (Figure 1).

**Figure 2:**
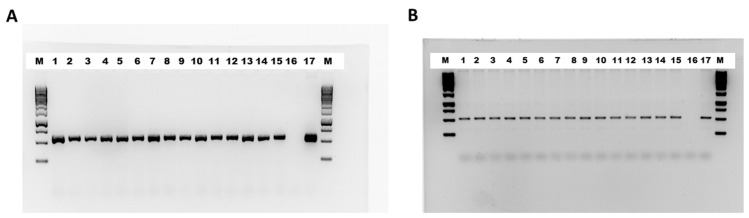
PCR results of *ScWS* (**A**) and *ScFAR* (**B**) of the transgenic lines. M, 1 kb DNA ladder from GeneRuler; L1–L15, transgenic lines; L16, wild type; L17, plasmid DNA.

Regarding WEs, 15 regenerated lines were confirmed to be transgenic by PCR analysis. All the transgenic lines showed integration of the *ScWS* (Figure 2A) and *ScFAR* genes (Figure 2B).

### 2.3. Expression Level of the Target Genes

**Figure 3:**
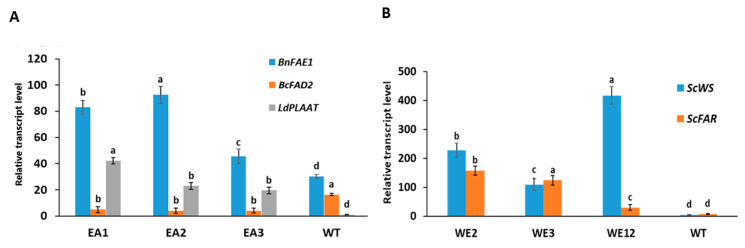
The gene expression levels of *BnFAE1*, *LdPLAAT* and *BcFAD2* in three erucic acid transgenic lines (EA1, EA2 and EA3) with reference to wild type (WT) (**A**)*,* and the expression levels of *ScWS* and *ScFAR* in three wax ester transgenic lines (WE2, WE3 and WE12) in comparison with WT (**B**). Results are means of three biological replicates for each line and three technical replicates per biological replicate. Bars followed by different letters indicate significant differences at *p* = 0.05.

Relative transcript levels of EA and WE genes for three transgenic lines along with the wild type were analyzed using qRT-PCR, and the results are presented in Figure 3. Compared to the transcript level in the wild type, higher levels of expression were observed for *BnFAE1* and *LdPLAAT*, while the expression of *BcFAD2* was downregulated in the transgenic lines (Figure 3A). A certain expression level in the *BnFAE1* gene was also detected in the wild type, and this is likely due to the presence of the endogenous *FAE1* genes in carinata. In the case of WE genes, the highest level of *ScWS* transcript was found in the WE12 line and the transcript level of *ScFAR* was higher for WE2, followed by WE3 (Figure 3B).

### 2.4. Fatty Acid Profile

The fatty acid profile results are presented in Table 2. A significant increase in C22:1 was observed ranging from 19% to 29% in the first three transgenic lines (EA1–EA3) in comparison with the wild type. The percentage of C18:1 tended to increase in all transgenic lines but with a significant increase only in two lines (EA4 and EA6) against the wild type. Meanwhile, all the transgenic lines produced a significantly lower percentage of C18:3 compared to the wild type.

### 2.5. WE Content

**Figure 4:**
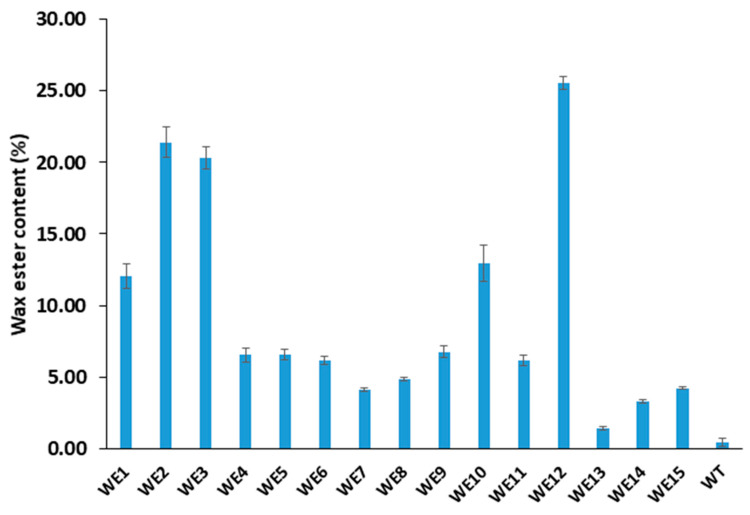
Wax ester contents of pooled seeds of 15 transgenic lines as determined by GC. Standard deviation and means of three biological replicates are shown (*n* = 3).

The WE contents of pooled seeds for the transgenic lines analyzed by GC are presented in Figure 4. The maximum WE content (25.6% of the neutral lipid fraction) was obtained from the line WE12, followed by WE2 and WE3, which contained 21.4% and 20.3% WE, respectively. Five transgenic lines (WE1, WE2, WE3, WE10 and WE12) that showed the highest WE contents (i.e., WE > 10%) were selected for single seed WE content analysis. As shown in Table 3, the trend of WE contents in these selected transgenic lines from the single seed analysis was similar to the result of pooled seed analysis, in which WE12 showed a significantly higher WE content (24.3%), followed by WE2 (14.8%) and WE3 (13.8%). There were, however, relatively larger differences in WE content between the pooled and individual seed analysis in the transgenic lines WE2 and WE3, while such differences were minimal in the W12 and WE10 transgenic lines. This is probably associated with the number of transgene copies and the position effect of transgene integration among the transgenic lines.

### 2.6. WE Content and Species Determined by nanoESI-MS/MS

The total WE contents of 15 transgenic lines analyzed by nanoESI-MS/MS ranged from 307 to 21,744 nmol/g seed (Figure S1), showing a similar trend with the results of the top four highest amounts of WE that were obtained by GC.

**Figure 5:**
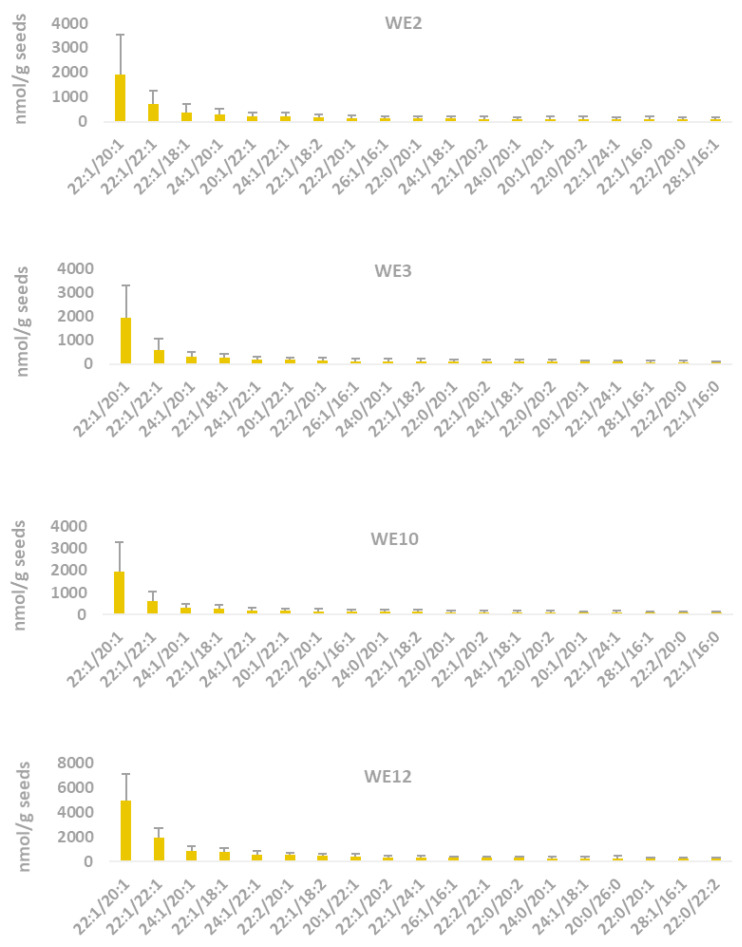
The 20 most abundant wax ester species in four transgenic lines analyzed by nanoESI-MS/MS. The results were means of four individual seeds of T_0_ plants and standard deviation is shown (*n* = 4).

The most dominating WE species in the top four transgenic lines was 22:1/20:1, followed by 22:1/22:1 (Table S1). For instance, the transgenic line WE12 produced WE species with the largest amounts of 22:1/20:1 and 22:1/22:1, accounting for ca. 23% and 9% of the total WE content, respectively (Figure 5).

### 2.7. Transgenic Lines Grown in Biotron

The T_0_ transgenic lines grew well in the biotron with controlled environmental conditions. The results of the seed-related traits of the transgenic lines along with the wild type are presented in Table 4. Since the data were collected from the T_0_ generation with only one plant per transgenic line, the results are considered very preliminary and provide only an indication of the traits presented. Some of the transgenic lines with high EA content tended to have a reduced thousand-seed weight (TSW) compared to the wild type, while the transgenic lines showed either higher or lower numbers of seeds per pod compared to the wild type. In the case of the WE lines, they tended to have higher or similar TSW in comparison to the wild type.

## 3. Discussion

Carinata is currently becoming the most demanded crop for delivering bio-industrial oil feedstock due to its high EA content [35]. In addition, the food versus fuel debate is not an issue of concern for carinata, unlike its counterpart crop, rapeseed, and it also has the ability to grow in marginal or drought-prone areas where other food crops fail. The increasing demand for the growing of carinata commercially as a bioenergy crop, such as biofuel or biodiesel, has made it an attractive renewable bioenergy source [36]. Although carinata contains a relatively high amount of EA, it is highly desirable to further increase the EA level to improve production efficiency. With each 10% increase in EA content, the cost of its purification from the seed oil will be reduced by half.

Genetic modification for increasing EA levels in oilseed crops has been shown to be a highly efficient approach. Transformation protocols have been developed in several oilseed species [37,38,39], which facilitate genetic modification of these crops. Extensive efforts have been made to increase EA in the oilseed crop, crambe (*C. abyssinica*) [31], while such efforts have been low in carinata. Mietkiewska et al. [28] demonstrated the possibility of increasing C22:1 and C18:1 by 16% and 10%, respectively, through the silencing of *FAD2* by RNAi in carinata, while Jadhav et al. [27] showed an increase in EA by 6–15% and 5–19% by co-suppression and antisense repression of *FAD2* in carinata, respectively. The increases in EA levels obtained in these studies are limited, likely due to the regulation of a single target gene, namely *FAD2*.

In other oilseed crops, more key genes involved in EA biosynthesis have been regulated using multiple gene constructs to increase the EA content [31,40]. Due to the inefficiency of the endogenous *LPAAT* genes in brassicas incorporating EA on the *sn*-2 position of the glycerol backbone, *LPAAT* genes from other plant species have been inserted into some brassica species to enhance EA production in seeds. For instance, the *LaLPAAT* gene from *Lunaria annua* and *LdLPLAAT* gene from *Limnathes douglasii* have been introduced into rapeseed, but the total EA level was not changed significantly [41]. This is likely due to the increase in EA at the *sn*-2 position being re-compensated by the decrease in EA at the *sn*-1 and *sn*-3 positions and the increase in EA by *LdPLAAT* being redistributed at the three hydroxyl positions of glycerol [41,42]. In another study by Nath et al. [30], when *BnFAE1* and *LdLPLAAT* were simultaneously introduced into rapeseed, EA was increased by 16%. This increment could be more if the apparent completion of C18:1 desaturation by *FAD2* is minimized, and this has been proved to be true in crambe, in which *LdPLAAT*, *BnFAE1* and *CaFAD2*-RNAi assembled in the same vector were introduced simultaneously and expressed [31]. In the present study, we used the same vector as for crambe to produce high EA carinata transgenic lines and obtained the transgenic lines expressing all three genes with an increase in EA of up to 29% and oleic acid of up to 113%. The overexpression of the *BnFAE1* and *LdLPAAT* genes accompanied by the downregulation of the *CaFAD2* gene, confirmed by the qRT-PCR results, was apparently associated with increased levels of oleic acid and EA in the transgenic lines EA1, EA2 and EA3. One should bear in mind that the *FAD2* sequence on the transformation construct used is from crambe but it shares 83% sequence homology with the carinata *FAD2* gene.

Currently, large-scale WE production for industrial applications is conducted mainly by chemical processes or lipases using petroleum or plant residue, which is becoming more and more expensive, complex and not environmentally friendly [43,44]. Although lipase-based synthesis of WEs is more environmentally friendly than the conventional chemical method, it has its own limitations in terms of its long processing time, making it more expensive, in addition to the use of hazardous reagents and the release of chemical waste [45,46]. Thus, metabolic engineering of oilseed crops has become an attractive strategy for renewable, sustainable and environmentally friendly production of WEs for industrial applications. WE production in plants is usually accomplished through the expression of the jojoba wax synthase and acyl-CoA reductase genes along with other important genes involved in oil biosynthesis [4], and this has been demonstrated in some oilseed crops [20,34,47]. In this study, we were able to obtain carinata transgenic lines containing WE content that is up to 25.6% of the total seed oil. The most abundant wax ester species was 22:1/20:1, followed by 22:1/22:1. It has been reported earlier that the most abundant WE species was 22:1/22:1, followed by 22:1/20:1, in transgenic carinata lines [20]. This difference is likely due to the background difference in initial carinata genotypes used for genetic transformation. In the latter case, a high EA transgenic line was used for genetic transformation to generate transgenic lines with WE, while in this study, a released carinata variety was used as the starting material for genetic modification.

The preliminary growth data of the transgenic lines with high EA content and the WE lines grown in bioton did not show a clear trend in the seed trait-related parameters as compared to the wild type. Such an evaluation must be carried out for a number of generations in order to make any meaningful conclusions about the performance of the transgenic lines.

## 4. Materials and Methods

### 4.1. Plant Material and In Vitro Growth Conditions

The seeds of carinata cultivar ‘*Derash*’, kindly provided by the Ethiopian Oilseeds Breeding Program of Holetta Agricultural Research Center, were used in this study. All in vitro cultures from seed germination to rooting were maintained in a growth chamber with a photoperiod of 16 h at 33 µmol m^−2^ s^−1^ and 21/18 °C (day/night).

### 4.2. Transformation Vectors

Two binary vectors, pWatergate4G and pBinGlyRed, were used for high erucic acid and wax ester transformation experiments of carinata, respectively. The pWatergate4G vector harbors the fatty acid elongase gene (*BnFAE1*) from *B. napus*, the lysophosphatidic acid acyltransferase gene (*LdLPAAT*) from *Limnathes douglasii* [30] and the RNAi-silenced fatty acid desaturase gene (*CaFAD2*-RNAi) from *Crambe abyssinica*, as described by Li et al. [31], and all the target genes are under the control of the seed-specific napin promoter. The pBinGlyRed vector harbored two genes, *ScFAR* and *ScWS*, derived from jojoba [20]. Both constructs harbored the *neomycin phosphotransferase II (NPTII)* gene as a selectable marker gene. The most virulent *Agrobacterium* strain, AGL-1, was used for plant transformation [48].

### 4.3. Transformation

#### 4.3.1. Seed Surface Sterilization

Seeds were surface sterilized with 12.5% calcium hypochlorite Ca(ClO)_2_ with 2–3 drops of Tween-20 for 10 min, followed by rinsing with sterilized water several times. Seeds were germinated in vitro on the full-strength MS [49] medium, supplemented with 20 g/L sucrose at pH 5.7 and 2.5 g/L Gelrite.

#### 4.3.2. Kanamycin Resistance Test

In order to optimize the concentration of the selective agent, a kanamycin resistance test was conducted prior to *Agrobacterium* transformation. The experiment consisted of five kanamycin concentrations (0, 15, 25, 35, 75 and 100 mg/L). The hypocotyls from the 5-day-old in vitro-grown seedlings were used in the test. The hypocotyls were cut into 3–5 mm length pieces and pre-cultured on a medium containing full-strength MS medium with MES, supplemented with 20 g/L sucrose, 2 mg/L BAP and 0.1 mg/L NAA, supplemented with 2.5 g/L Gelrite at pH 5.7 for 2 days. The hypocotyls were then moved to the same medium but with the addition of different concentrations of kanamycin. Twenty hypocotyls per Petri dish were used in the test. The experiment was repeated three times.

#### 4.3.3. *Agrobacterium*-Mediated Transformation

For transformation, the overnight-grown *Agrobacterium* suspension was pelleted by centrifugation at 4200 rpm for 10 min. The pellet was suspended in 10 mL of liquid MS20 medium (MS, 20 g/L sucrose, pH 5.7) at a concentration with an optical density of 0.5 at 600 nm (OD600). The pre-cultured explants were incubated in the *Agrobacterium* suspension for 10 min with gentle shaking. The explants were blotted on sterile filter paper to remove excess *Agrobacterium* suspension and then transferred to the same medium as the pre-culture. The cultures were maintained in the growth chamber as described above for 2 days in the dark.

After co-culture, the explants were rinsed with liquid MS20 medium, supplemented with cefotaxime (400 mg/L) and placed on the selective regeneration medium (CIMs25), supplemented with 2 mg/L BAP, 0.1 mg/L NAA and 25 mg/L kanamycin. After 30 days or two subcultures, the selection pressure was increased to 35 mg/L kanamycin in the MS20 medium supplemented with 1 mg/L BAP, 1 mg/L GA3 and 7 g/L agar at pH 5.7. The explants were transferred to a fresh selection medium every two weeks and maintained in the growth chamber, as stated above.

### 4.4. Confirmation of Transgenic Lines by PCR Analysis

Genomic DNA was extracted from the leaves of the putative transgenic lines along with the wild type using the CTAB method, as described by Aldrich and Cullis [50]. PCR analysis was conducted for the target genes using the gene-specific primers listed in Table 5. The PCR program consisted of initial denaturation at 95 °C for 1 min, followed by 30 cycles of denaturation at 95 °C for 10 s, primer annealing at 60–65 °C for 30 s, depending on the primer, and extension at 72 °C for 1 min. The PCR products were analyzed on a 1% (*w*/*v*) agarose gel.

### 4.5. Quantitative Real-Time PCR (qRT-PCR) Analysis

Total RNA was extracted from immature pods of carinata collected at 25–35 days after flowering using the RNase Plant Mini Kit (Qiagen, Hilden, Germany) following the manufacturer’s protocol. The primers for the target genes were selected from several sets of primers tested for their efficiency and specificity. *UBC21* was used as a reference gene to normalize gene expression, as demonstrated in *B. napus* [51]. The primers used are presented in Table 6. Each transgenic line, along with the wild type, was sampled with three biological replicates and three technical replicates for running qRT-PCR according to the standard method used in our lab, as described by Ivarson et al. [34].

### 4.6. Growth of Transgenic Lines in Biotron

In vitro rooted T_0_ transgenic lines along with the wild type were grown in biotron under a controlled environment with a 16 h photoperiod, 250 µmol m^−2^ s^−1^ light intensity, 21/18 °C temperature (day/light) and 65% humidity. The plants were watered regularly with normal management. Data on yield-related traits such as thousand-seed weight (TSW), number of seeds per pod and number of pods per plant were collected. After harvesting, the seeds were kept at 4 °C for further analysis.

### 4.7. Fatty Acid Profiling by Gas Chromatography (GC)

About 0.5 g of seeds from each transgenic line and wild type were weighed in triplicate and placed in a glass tube with a non-screw cap suited for crushing. Then, 3.75 mL of extraction solution consisting of methanol (MeOH) and chloroform (CHCl_3_) in the ratio of 2:1 (*v*/*v*) along with 1 mL of 0.15 M acetic acid was added to the tube. Seeds were then homogenized using an IKA^®^T18 basic (ULTRATURRAX^®^, IKA, Staufen, Germany). After homogenization, 1.25 mL of CHCl_3_ and 0.9 mL of H_2_O were added and mixed by vortexing for 30 s. The samples were centrifuged at 3000 rpm for 3 min. The lower phase was transferred to a new screw-cap tube and allowed to dry completely under a stream of nitrogen gas on a heated sand bed at 70 °C. The residue was dissolved in 100 µL of heptane (GC grade), methylated by adding 2 mL of methylation solution (2% H_2_SO_4_ in anhydrous methanol) and incubated at 90 °C for 1 h. The methylated samples containing fatty acid methyl esters (FAMEs) were allowed to cool down to room temperature before 1 mL of H_2_O and 0.75 mL of heptane (GC grade) were added to the samples. The samples were vortexed and centrifuged for 2 min at 2000 rpm, and 200 µL of the upper heptane phase containing FAMEs was transferred to a GC vial for analysis. The samples were finally analyzed on Agilent (Model 8860, Agilent, Solna, Sweden) GC equipped with a flame ionization detector (FID) and a WCOT Fused Silica capillary column (50 m × 0.32 mm) coated with CP-Wax 58 column with a split ratio of 10:1 and an oven program of 150 °C for 0.2 min, 4 °C/min to 210 °C, 10 °C/min to 250 °C and then holding at 250 °C for 5 min. The fatty acid profile was identified based on the retention times of peaks corresponding to their respective FAMEs with reference to a certified Me63 fatty acid methyl ester mixture (Larodan Fine Chemicals AB, Malmo, Sweden) as an external standard. Quantification of individual fatty acids was based on the area of their respective peaks (Figure S2).

### 4.8. Analysis of WE Content and Profile

#### 4.8.1. WE Analysis with GC

WEs were extracted from 10 pooled seed samples of the 15 transgenic lines in triplicate first, followed by single seed analysis of the transgenic lines and wild type, as described by Li et al. [31]. For the pooled samples, the seeds were homogenized with the extraction solution (3.75 mL of MeOH:CHCl_3_ (2:1 *v*/*v*) and 1 mL of 0.15 M acetic acid (HAc)) using an ultraturrax (IKA^®^ T18 basic, ULTRATURRAX^®^). After homogenization, 1.25 mL of CHCl_3_ and 1.25 mL of H_2_O were added and mixed by vortexing before centrifugation at 3000 rpm for 3 min. For the single seed analysis, the samples were homogenized in 1 mL of 0.15 M HAc using a mortar and pestle. After homogenization, 3.75 mL of MeOH:CHCl_3_ (2:1 *v*/*v*) was added, and the samples were then transferred to a new screw-capped glass tube for centrifugation. The lower CHCl_3_ phase was transferred to a new screw-cap tube and dried completely under a nitrogen stream. The residue was then re-suspended in 200 μL of CHCl_3_ for thin-layer chromatography (TLC) separation. For each sample, 30 μL was loaded on a silica gel TLC plate (20 × 20 cm, Merk, Darmstad, Germany) and developed in heptane/diethyl-ether (DEE)/HAc (90:10:1 *v*/*v*/*v*) for 10–15 min to separate the WEs and TAGs, and the process was visualized by exposure to iodine vapor. For GC analysis, the areas of the silica gel containing TAGs and WEs were scraped off separately by spraying the plate with H_2_O and collected in a screw-capped tube with the addition of 200 μL of methanol. The samples were dried under a N_2_ stream and methylated by adding 2 mL of methylation solution (2% H_2_SO_4_ in methanol) and incubated at 90 °C for 1 h. After methylation, 0.5 mL of heptane, 2 mL of H_2_O and an internal standard of 200 mM methyl-heptadecanoate (17:0-ME, Larodan, Solna, Sweden) were added and the solution was vortexed and centrifuged at 3000 rpm for 3 min. The upper heptane phase containing the methylated products was transferred to GC vials for GC analysis, as described by Zhu et al. [20]. The contents of TAG and WE were then calculated based on their peak areas.

#### 4.8.2. WE Profiling Analyzed by Nanoelectrospray Coupled with Tandem Mass Spectrometry (nanoESI-MS/MS)

WE content and molecular species profile in the seed oil extracts from the transgenic lines and wild type were obtained using the direct infusion nanoESI-MS/MS, as described by Iven et al. [52] with minor modifications. The seed oil of four seeds per transgenic line was extracted with 5 nmol heptadecanoyl-heptadecanoate (Nu-Chek Prep, Inc., Elysian, MN, USA), and purified WEs were obtained by TLC separation. The WE fractions were then analyzed by nanoESI-MS/MS.

### 4.9. Statistical Analysis

Data were subjected to ANOVA analysis using MINITAB version 18 (Minitab, LLC, State College, PA, USA) whenever applicable. Treatment mean comparison was made using the Tukey–Kramer method at the *p =* 0.05 level.

## 5. Conclusions

Our study has demonstrated the possibility of engineering carinata for high EA levels and wax ester production. In the case of EA, the use of a multigene construct that encompasses the two genes *BnFAE1* and *LdLPLAAT* along with the *RNAi*-silenced *CaFAD2* gene was found to be more effective for the enhancement of EA. We were able to obtain transgenic lines with up to 52.7% EA content through overexpression of *BnFAE1* and *LdLPLAAT* and downregulation of *CaFAD2*. Considering wax esters, transgenic lines produced WEs within the range of 8% to 25%, which showed the effectiveness of wax ester production from carinata. The above outputs imply that the bio-industrial oil quality of carinata can be enhanced by increasing EA levels using the gene stacking strategy and by introducing genes for producing neutral lipids like WEs.

## Figures and Tables

**Table 1:** Shoot regeneration from hypocotyls of *B. carinata* grown on the medium containing different concentrations of kanamycin.

Kanamycin Concentration (mg/L)	Shoot Regeneration % (Mean ± SD)
0	20.0 ± 0.0 ^a^
15	18.7 ± 0.6 ^a^
25	14.0 ± 2.0 ^b^
35	11.3 ± 1.2 ^b^
50	4.7 ± 1.5 ^c^
75	0.7 ± 0.6 ^d^
100	0.0 ± 0.0 ^d^

Note: Each treatment contained 20 explants and was repeated three times. Means followed by different letters indicate significant differences at *p* = 0.05 (*n* = 3).

**Table 2:** The relative contents of four major fatty acids in the six transgenic lines for high erucic acid (EA) types and wild type (WT).

Lines	C18:1	C18:2	C18:3	C22:1
EA1	15.9 ± 2.2 ^ab^	5.7 ± 0.7 ^b^	8.2 ± 1.1 ^b^	52.7 ± 2.1 ^a^
EA2	17.6 ± 0.8 ^ab^	5.9 ± 1.3 ^b^	8.4 ± 0.8 ^b^	50.8 ± 2.5 ^a^
EA3	18.5 ± 3.2 ^ab^	6.9 ± 2.1 ^ab^	8.1 ± 1.6 ^b^	48.5 ± 3.3 ^ab^
EA4	21.8 ± 1.8 ^a^	6.6 ± 1.6 ^ab^	9.4 ± 0.7 ^b^	44.5 ± 0.6 ^bc^
EA5	19.5 ± 6.2 ^ab^	7.9 ± 3.3 ^ab^	10.1 ± 3.0 ^b^	43.4 ±2.0 ^bc^
EA6	22.8 ± 4.0 ^a^	5.59 ± 1.4 ^b^	9.8 ± 1.6 ^b^	43.1 ± 1.0 ^bc^
WT	10.7 ± 0.5 ^b^	11.3 ± 0.2 ^a^	15.1 ± 1.0 ^a^	40.9 ± 0.9 ^c^

Note: Means and standard deviation of three biological replicates for each line are shown. Means followed by different letters in the same column indicate significant differences at *p* = 0.05 (*n* = 3).

**Table 3:** Wax ester content in single seeds of the six most WE-abundant transgenic lines in comparison with those from the pooled seeds of the same lines in T_0_.

Transgenic Line	Single Seed WE (%)	Pooled Seed WE (%)
WE1	8.1 ± 2.3 ^b^	12.08 ± 0.85 ^c^
WE2	14.8 ± 2.5 ^b^	21.40 ± 1.1 ^b^
WE3	13.8 ± 5.3 ^b^	20.34 ± 0.8 ^b^
WE10	10.9 ± 4.0 ^b^	12.94 ± 1.2 ^c^
WE12	24.3 ± 4.7 ^a^	25.56 ± 0.5 ^a^

Note: Means and standard deviation of three biological replicates for each line are shown. Means followed by different letters in the same column indicate significant differences at *p* = 0.05. For single seed analysis, means and standard deviation of 20 seeds were analyzed per transgenic line and 3 biological replicates were performed for the pooled seed analysis.

**Table 4:** Seed-related traits of the transgenic lines in T_0_ generation grown in biotron.

Line	TSW (g)	No. of Pods per Plant	No. of Seeds per Pod
EA-1	4.52	272	7.1 ± 1.1 ^d^
EA-2	4.32	274	6.1 ± 1.0 ^e^
EA-3	4.42	276	5.2 ± 1.1 ^f^
EA-4	4.12	238	9.0 ± 0.6 ^b^
EA-5	4.24	230	8.9 ± 1.1 ^b^
EA-6	4.38	236	9.9 ± 1.3 ^a^
WT	5.01	259	8.0 ± 0.9 ^c^
WE-1	5.6	245	7.1 ± 1.1 ^ef^
WE-2	5.4	238	8.1 ± 0.9 ^bcd^
WE-3	5.6	240	9.0 ±1.3 ^ab^
WE-4	5.4	210	8.6 ± 1.0 ^bc^
WE-5	5.4	185	6.0 ± 1.0 ^gh^
WE-6	5.7	180	8.0 ± 1.0 ^cde^
WE-7	5.4	265	5.9 ± 0.9 ^gh^
WE-8	5.4	270	6.1 ± 1.0 ^fg^
WE-9	5.6	261	5.17 ± 1.09 ^h^
WE-10	5.3	280	5.9 ± 1.0 ^gh^
WE-11	5.3	295	8.9 ± 1.1 ^bc^
WE-12	5.4	310	9.9 ± 1.30 ^a^
WE-13	5.6	258	8.1 ± 1.4 ^bcd^
WE-14	5.4	268	8.1 ± 0.9 ^bcd^
WE-15	5.5	260	9.0 ± 1.2 ^b^
WT	5.1	256	7.6 ± 1.0 ^de^

Note: EA, erucic acid; WE, wax ester; WT, wild type; TSW, thousand-seed weight. Means and standard deviation of three biological replicates for each line are shown. Means followed by different letters indicate significant differences at *p* = 0.05 (*n* = 30).

**Table 5:** The sequence of the primers used for PCR analysis.

Gene	Forward Primers (5′-3′)	Reverse Primers (5′-3′)
*BnFAE1*	AATGCGTTGGTGGAAGGTAG	TGTGTAGCTCATGTCCTGGC
*LdPLAAT*	GTGGTTTTTGAGACGCAGGT	TAGACTGAATAGCCGCAGCC
*ScFAR*	AGGCATTAGGGGAGATGCTT	CCTTGAACCATTGGCAGAAT
*ScWS*	CTCTTCGCCTTTCATCTTGG	AACACAAAGAACCCCGTCAC

**Table 6:** The sequences of the primers used for qRT-PCR analysis.

Gene	Forward Primers (5′-3′)	Reverse Primers (5′-3′)
*BnFAE1*	CCTCCCCGGAAGACTTTTG	CATGCTTGAGTTCACCACAAG
*BcFAD2*	CCGTGAACGTCTCCAGATAT	CGTTGACTATCAGAAGCGGA
*LdPLAAT*	AAGTAAACGCCCATCTCTCG	GGCTGCGGCTATTCAGTCTA
*ScFAR*	CTCCTCCTTCTCCACCTTCC	CCTTGAACCATTGGCAGAAT
*ScWS*	CTCTTCGCCTTTCATCTTGG	CTCGATGTGTTCCCAAACCT
*AtUBC21*	TGCGACTCAGGGAATCTTCT	TCATCCTTTCTTAGGCATAGCG

## Data Availability

All data analyzed during this study are included in this published article.

## Supplementary Materials

The following supporting information can be downloaded at: https://www.mdpi.com/article/10.3390/ijms25126322/s1.

## References

[1] Durrett T.P., Benning C., Ohlrogge J. (2008). Plant triacylglycerols as feedstocks for the production of biofuels. Plant J..

[2] Lu C., Napier J., Clemente T., Cahoon E. (2011). New frontiers in oilseed biotechnology: Meeting the growing global demand for vegetable oils for food, feed, biofuel, and industrial uses. Curr. Opin. Biotechnol..

[3] Mnzava N., Schippers R., van der Vossen H.A.M., Mkamilo G.S. (2007). Brassica carinata A. Braun. [Internet] Record from PROTA4U.

[4] Taylor D.C., Falk K.C., Palmer C.D., Hammerlindl J., Babic V., Mietkiewska E., Jadhav A., Marillia E.-F., Francis T., Hoffman T. (2010). *Brassica carinata*—A new molecular farming platform for delivering bio-industrial oil feedstocks: Case studies of genetic modifications to improve very long-chain fatty acid and oil content in seeds. Biofuels Bioprod. Biorefin..

[5] Roslinsky V., Falk K.C., Gaebelein R., Mason A.S., Eynck C. (2021). Development of *B. carinata* with super-high erucic acid content through interspecific hybridization. Theor. Appl. Genet..

[6] Derksen J.T., Cuperus F.P., Kolster P. (1995). Paints and coatings from renewable resources. Ind. Crops Prod..

[7] Leonard E.C. (1992). High-erucic vegetable oils. Ind. Crops Prod..

[8] McVetty P.B., Scarth R. (2002). Breeding for improved oil quality in *Brassica oilseed* species. J. Crops Prod..

[9] Semenov V., Semenova D., Slipushenko V. (2006). Calculation of the high heat value of biofuels. Chem. Technol. Fuels Oils.

[10] Ghanevati M., Jaworski J.G. (2002). Engineering and mechanistic studies of the Arabidopsis FAE1 β-ketoacyl-CoA synthase, FAE1 KCS. Eur. J. Biochem..

[11] Sanyal A., Pinochet X., Merrien A., Laustriat M., Decocq G., Fine F. (2015). Erucic acid rapeseed: 1. Prospects of improvements. OCL.

[12] Becker H., Löptien H., Röbbelen G., Gómez-Campo C. (1999). Breeding: An overview. Biology of Brassica Coenospecies.

[13] Röbbelen G. (1980). Biosynthesis of seed oil and breeding for improved oil quality of rapeseed. Brassica Crops and Wild Allies.

[14] Warwick S., Gugel R., McDonald T., Falk K. (2006). Genetic variation and agronomic potential of Ethiopian mustard (*Brassica carinata*) in western Canada. Genet. Resour. Crop Evol..

[15] Sasongko N.D., Möllers C. (2005). Toward increasing erucic acid content in oilseed rape (*Brassica napus* L.) through the combination with genes for high oleic acid. J. Am. Oil Chem. Soc..

[16] Zanetti F., Mosca G., Rampin E., Vamerali T. (2012). Adaptability and sustainable management of high-erucic Brassicaceae in Mediterranean environment. Oilseeds.

[17] George S., Seepaul R., Geller D., Dwivedi P., DiLorenzo N., Altman R., Coppola E., Miller S.A., Bennett R., Johnston G. (2021). A regional inter-disciplinary partnership focusing on the development of a carinata-centered bioeconomy. GCB Bioenergy.

[18] Domergue F., Miklaszewska M. (2022). The production of wax esters in transgenic plants: Towards a sustainable source of bio-lubricants. J. Exp. Bot..

[19] Miwa T.K. (1971). Jojoba oil wax esters and derived fatty acids and alcohols: Gas chromatographic analyses. J. Am. Oil Chem. Soc..

[20] Zhu L.-H., Krens F., Smith M.A., Li X., Qi W., Van Loo E.N., Iven T., Feussner I., Nazarenus T.J., Huai D. (2016). Dedicated industrial oilseed crops as metabolic engineering platforms for sustainable industrial feedstock production. Sci. Rep..

[21] Wu X.-Y., Moreau R.A., Stumpf P.K. (1981). Studies of biosynthesis of waxes by developing jojoba seed: III. Biosynthesis of wax esters from Acyl-CoA and long chain alcohols. Lipids.

[22] Vanhercke T., Wood C.C., Stymne S., Singh S.P., Green A.G. (2013). Metabolic engineering of plant oils and waxes for use as industrial feedstocks. Plant Biotechnol. J..

[23] Babic V., Datla R., Scoles G., Keller W. (1998). Development of an efficient Agrobacterium-mediated transformation system for Brassica carinata. Plant Cell Rep..

[24] Narasimhulu S.B., Kirti P.B., Mohapatra T., Prakash S., Chopra V.L. (1992). Shoot regeneration in stem explants and its amenability to Agrobacterium tumefaciens mediated gene transfer in *Brassica carinata*. Plant Cell Rep..

[25] Palmer C.E., Keller W.A., Khachatourians G.C. (2002). Transgenic oilseed Brassicas. Transgenic Plants and Crops.

[26] Poulsen G. (1996). Genetic transformation of Brassica. Plant Breed..

[27] Jadhav A., Katavic V., Marillia E.-F., Giblin E.M., Barton D.L., Kumar A., Sonntag C., Babic V., Keller W.A., Taylor D.C. (2005). Increased levels of erucic acid in *Brassica carinata* by co-suppression and antisense repression of the endogenous *FAD2* gene. Metab. Eng..

[28] Mietkiewska E., Hoffman T.L., Brost J.M., Giblin E.M., Barton D.L., Francis T., Zhang Y., Taylor D.C. (2008). Hairpin-RNA mediated silencing of endogenous *FAD2* gene combined with heterologous expression of *Crambe abyssinica FAE* gene causes a dramatic increase in the level of erucic acid in transgenic *Brassica carinata* seeds. Mol. Breed..

[29] Kuo T.M., Gardner H.W. (2002). Lipases: Structure, function, and properties. Lipid Biotechnology.

[30] Nath N.K., Wilmer J.A., Wallington E.J., Becker H.C., Möllers C. (2009). Increasing erucic acid content through combination of endogenouslow polyunsaturated fatty acids alleles withLd-LPAAT + Bn-fae1 transgenesin rapeseed (*Brassica napus* L.). Theor. Appl. Genet..

[31] Li X., van Loo E.N., Gruber J., Fan J., Guan R., Frentzen M., Stymne S., Zhu L.H. (2012). Development of ultra-high erucic acid oil in the industrial oil crop *Crambe abyssinica*. Plant Biotechnol. J..

[32] Li X., Guan R., Fan J., Zhu L.-H. (2019). Development of Industrial Oil Crop *Crambe abyssinica* for Wax Ester Production through Metabolic Engineering and Cross Breeding. Plant Cell Physiol..

[33] Lardizabal K.D., Metz J.G., Sakamoto T., Hutton W.C., Pollard M.R., Lassner M.W. (2000). Purification of a jojoba embryo wax synthase, cloning of its cDNA, and production of high levels of wax in seeds of transgenic Arabidopsis. Plant Physiol..

[34] Ivarson E., Iven T., Sturtevant D., Ahlman A., Cai Y., Chapman K., Feussner I., Zhu L.-H. (2017). Production of wax esters in the wild oil species *Lepidium campestre*. Ind. Crops Prod..

[35] Taylor D.C., Smith M.A., Fobert P., Mietkiewaska E., Weselake R.J. (2011). Metabolic engineering of higher plants to produce bio-industrial oils. Compr. Biotechnol..

[36] Marillia E.-F., Francis T., Falk K.C., Smith M., Taylor D.C. (2014). Palliser’s promise: Brassica carinata, an emerging western Canadian crop for delivery of new bio-industrial oil feedstocks. Biocatal. Agric. Biotechnol..

[37] Ivarson E., Ahlman A., Li X., Zhu L.-H. (2013). Development of an efficient regeneration and transformation method for the new potential oilseed crop *Lepidium campestre*. BMC Plant Biol..

[38] Li X., Ahlman A., Yan X., Lindgren H., Zhu L.-H. (2010). Genetic transformation of the oilseed crop *Crambe abyssinica*. Plant Cell Tissue Organ Cult..

[39] Yadav R.C., Singh D. (2013). Genetic transformation in oilseed brassicas-A. Indian J. Agric. Sci..

[40] Wang P., Xiong X., Zhang X., Wu G., Liu F. (2022). A review of erucic acid production in *Brassicaceae oilseeds*: Progress and prospects for the genetic engineering of high and low-erucic acid rapeseeds (*Brassica napus*). Front. Plant Sci..

[41] Lassner M.W., Levering C.K., Davies H.M., Knutzon D.S. (1995). Lysophosphatidic acid acyltransferase from meadowfoam mediates insertion of erucic acid at the sn-2 position of triacylglycerol in transgenic rapeseed oil. Plant Physiol..

[42] Brough C.L., Coventry J.M., Christie W.W., Kroon J.T.M., Brown A.R., Barsby T.L., Slabas A.R. (1996). Towards the genetic engineering of triacylglycerols of defined fatty acid composition: Major changes in erucic acid content at the sn-2 position affected by the introduction of a 1-acyl-sn-glycerol-3-phosphate acyltransferase from *Limnanthes douglasii* into oil seed rape. Mol. Breed..

[43] Lokotsch W., Lang S., Möbius D., Wagner F. (1996). Biocatalytical synthesis and monolayer studies of multiple hydroxylated wax esters. J. Am. Oil Chem. Soc..

[44] Nguyen D.H., Raffa G., Morin Y., Desset S., Capet F., Nardello-Rataj V., Dumeignil F., Gauvin R.M. (2017). Solvent-and base-free synthesis of wax esters from fatty acid methyl esters by consecutive one-pot, two-step catalysis. Green Chem..

[45] Hagström Å.K., Wang H.-L., Liénard M.A., Lassance J.-M., Johansson T., Löfstedt C. (2013). A moth pheromone brewery: Production of (Z)-11-hexadecenol by heterologous co-expression of two biosynthetic genes from a noctuid moth in a yeast cell factory. Microb. Cell Factories.

[46] Munkajohnpong P., Kesornpun C., Buttranon S., Jaroensuk J., Weeranoppanant N., Chaiyen P. (2020). Fatty alcohol production: An opportunity of bioprocess. Biofuels Bioprod. Biorefin..

[47] Bansal S., Durrett T.P. (2016). Camelina sativa: An ideal platform for the metabolic engineering and field production of industrial lipids. Biochimie.

[48] Lazo G.R., Stein P.A., Ludwig R.A. (1991). A DNA Transformation-Competent *Arabidopsis* Genomic Library in *Agrobacterium*. Bio-Technology.

[49] Murashige T., Skoog F. (1962). A revised medium for rapid growth and bio assays with tobacco tissue cultures. Physiol. Plant..

[50] Aldrich J., Cullis C. (1993). RAPD analysis in flax: Optimization of yield and reproducibility sing Klen Taq1 DNA polymerase, Chelex 100, and gel purification of genomic DNA. Plant Mol. Biol. Rep..

[51] Han J., Lühs W., Sonntag K., Zähringer U., Borchardt D.S., Wolter F.P., Heinz E., Frentzen M. (2001). Functional characterization of â-ketoacyl-CoA synthase genes from *Brassica napus* L. Plant Mol. Biol..

[52] Iven T., Herrfurth C., Hornung E., Heilmann M., Hofvander P., Stymne S., Zhu L.H., Feussner I. (2013). Wax ester profiling of seed oil by nano-electrospray ionization tandem mass spectrometry. Plant Methods.

