# Supplementary material for: Enhancing Erucic Acid and Wax Ester Production in Brassica carinata through Metabolic Engineering for Industrial Applications
Source: Int J Mol Sci. 2024 Jun 7;25(12):6322. doi: 10.3390/ijms25126322 (PMC11203470; doi:10.3390/ijms25126322)
Supplement: Supplementary file 1 [file ijms-25-06322-s001.zip › ijms-3044686-supplementary.pdf]

Supplementary data file

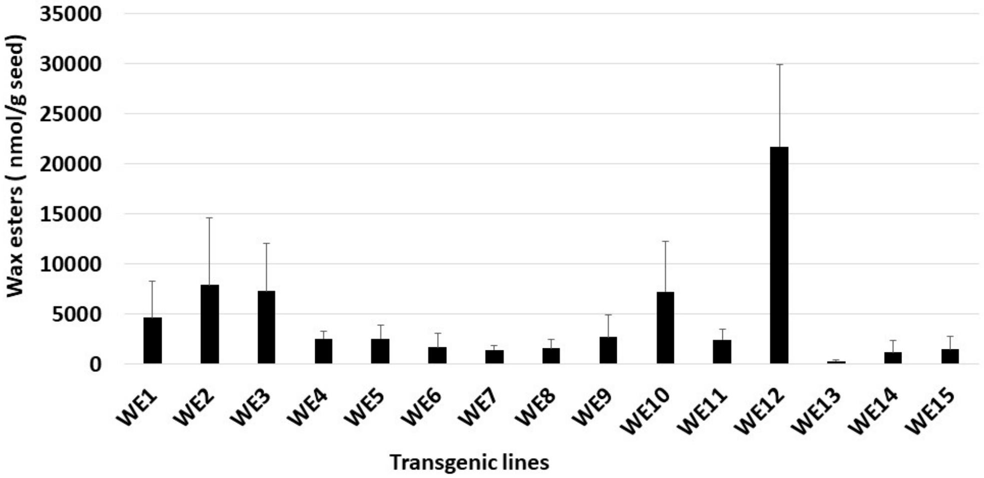

Figure S1. The total wax ester contents of 15 transgenic lines harboring *ScWS/ScFAR* analyzed by nanoESI-MS/MS.

Wild type

Fatty acid	Retention time (min)
C18:1	8,381
C18:2	9,123
C18:3	10,211
C22:1	15,402

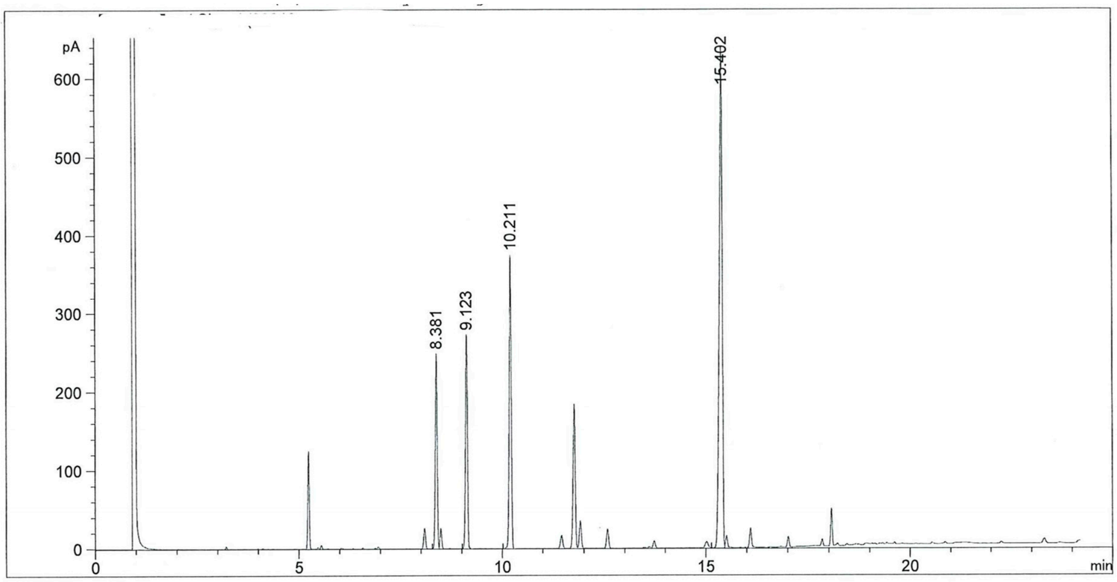

EA1

Fatty acid	Retention time (min)
C18:1	8,388
C18:2	9,117
C18:3	10,199
C22:1	15,414

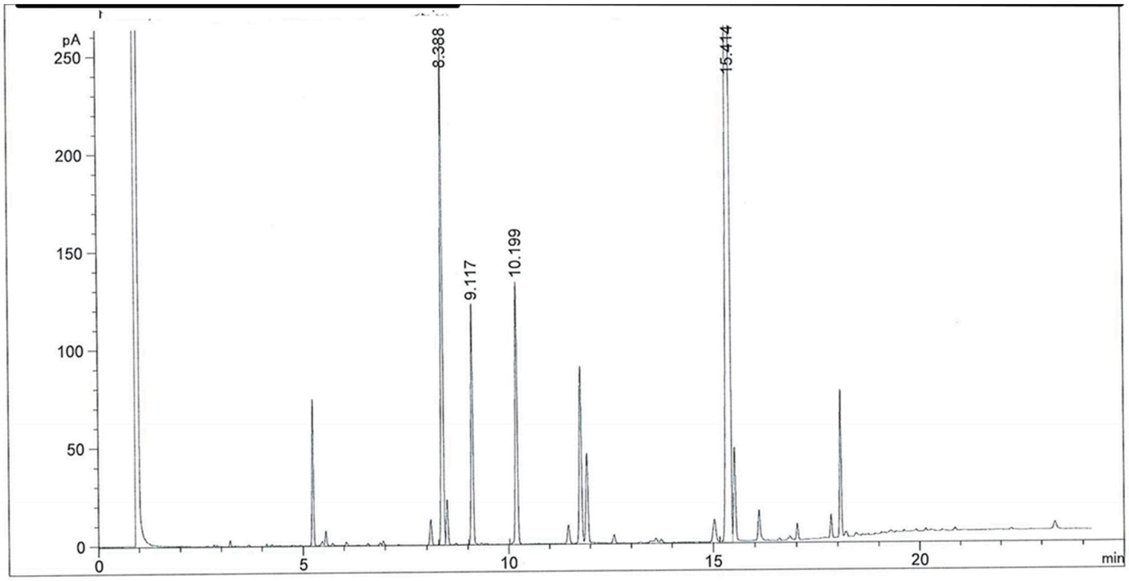

EA2

Fatty acid	Retention time (min)
C18:1	8385
C18:2	9,112
C18:3	10,194
C22:1	15,383

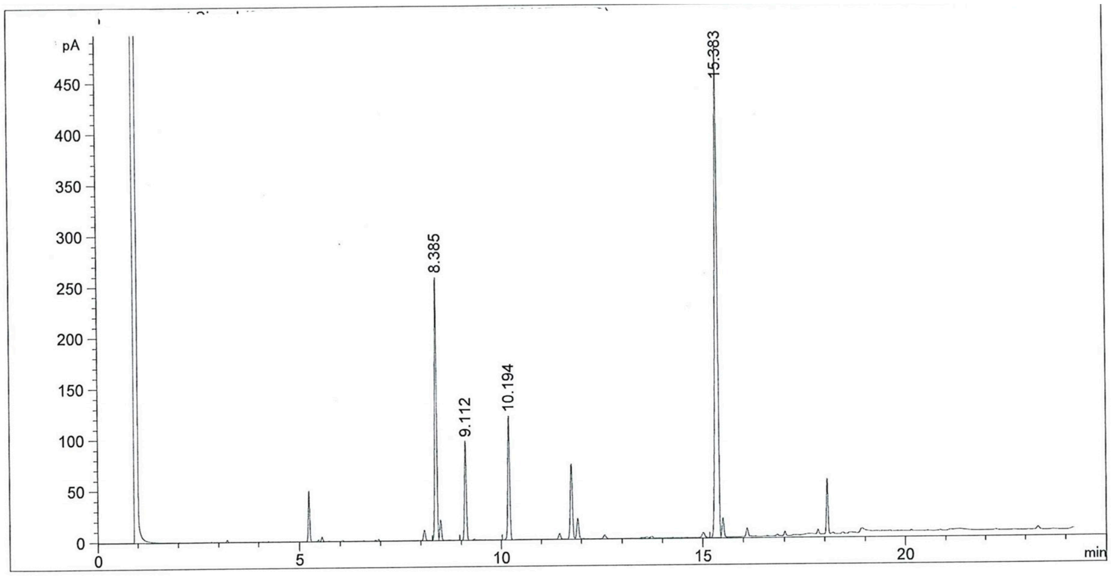

EA3

Fatty acid	Retention time (min)
C18:1	8,387
C18:2	9,11
C18:3	10,19
C22:1	15,38

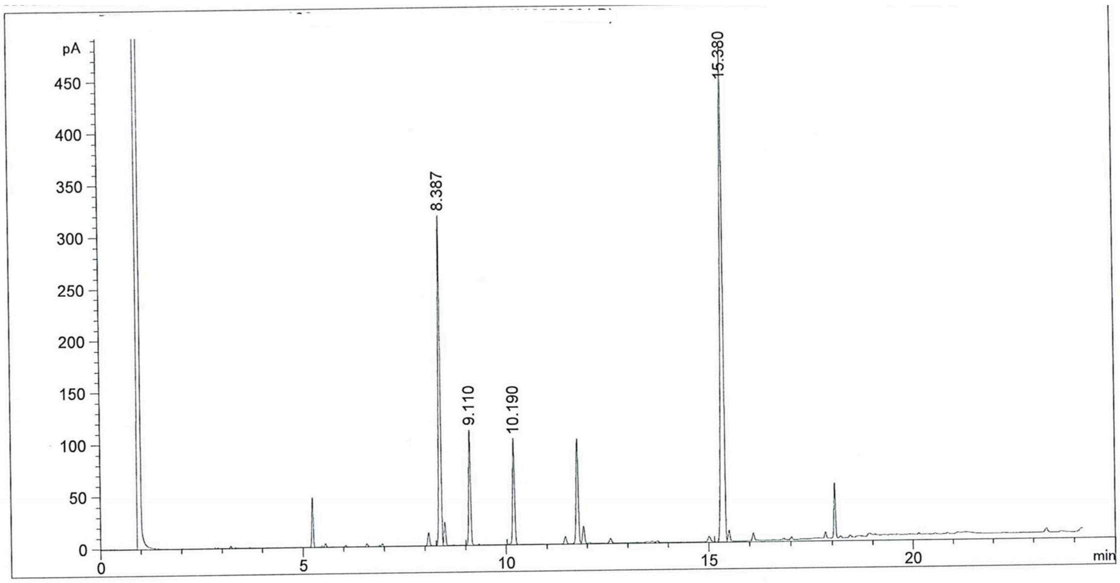

EA4

Fatty acid	Retention time (min)
C18:1	8,4
C18:2	9,133
C18:3	10,213
C22:1	15,381

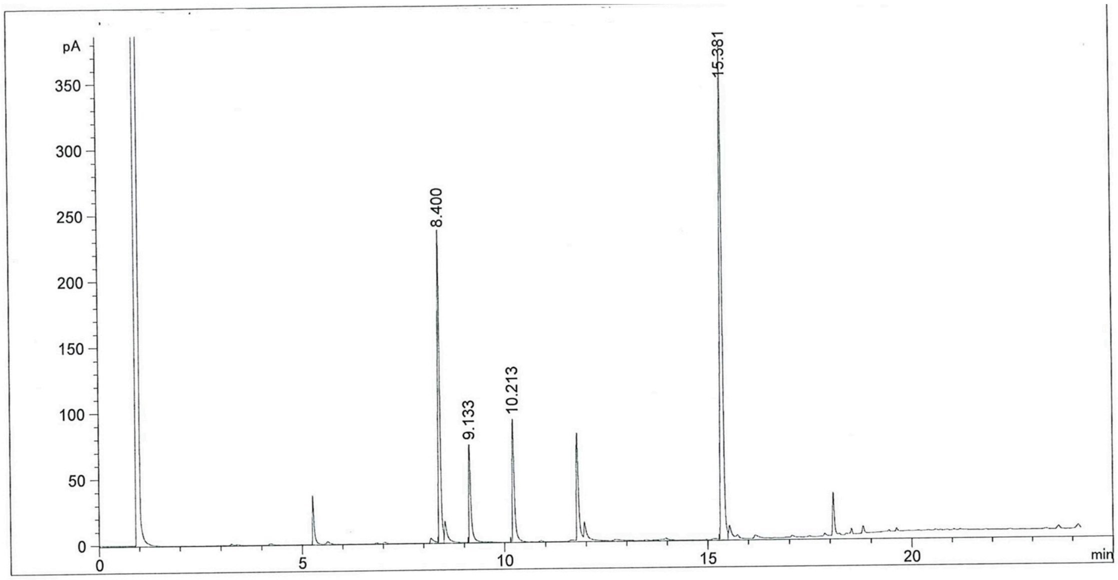

EA5

Fatty acid	Retention time (min)
C18:1	8,373
C18:2	9,108

C18:3	10,187
C22:1	15,35

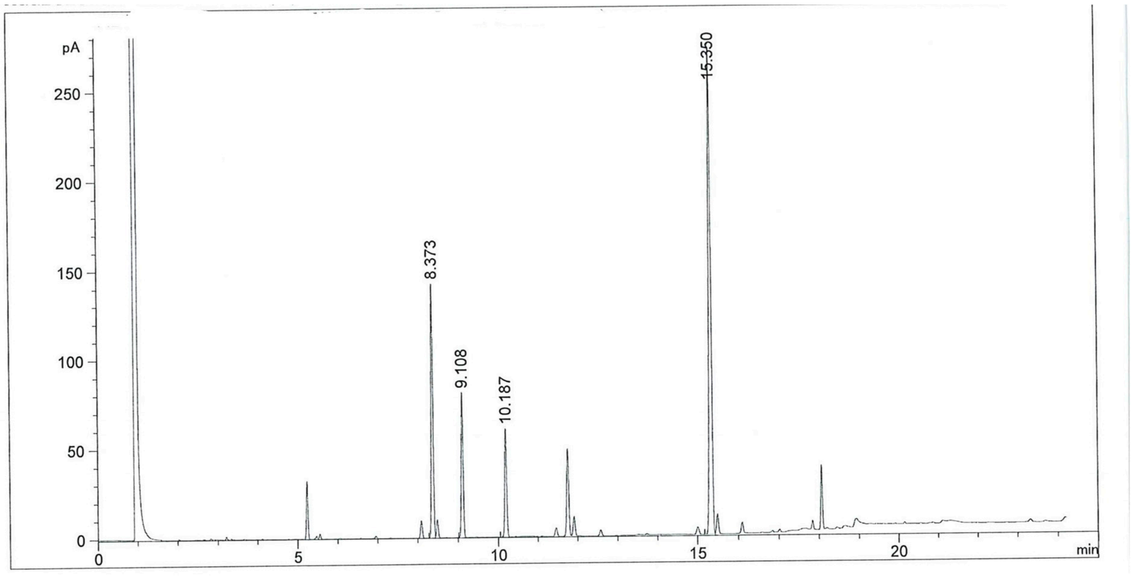

EA6

Fatty acid	Retention time (min)
C18:1	8,393
C18:2	9,11
C18:3	10,197
C22:1	15,386

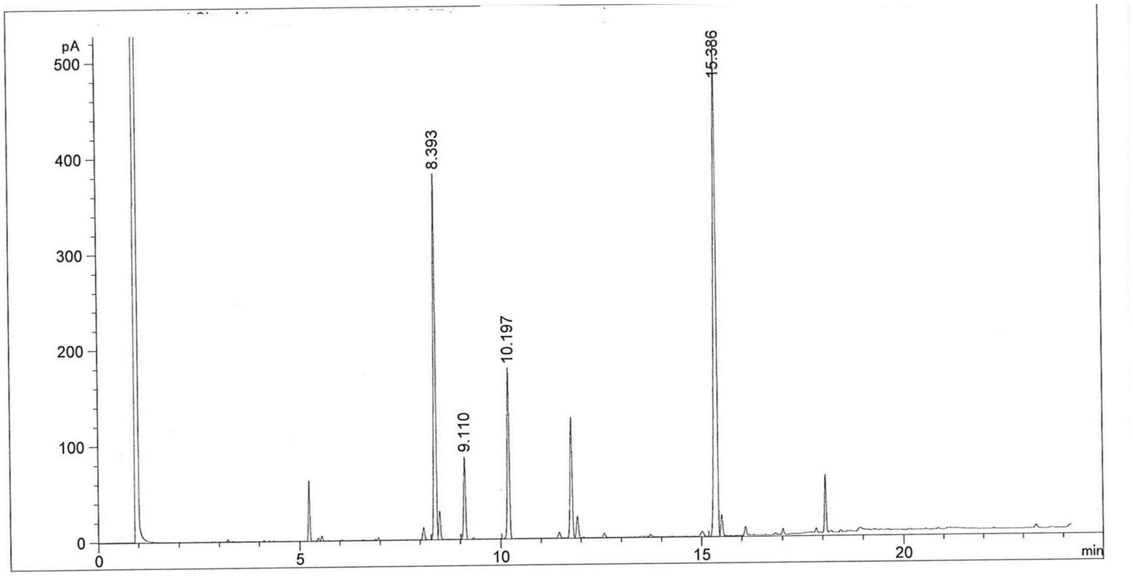

Figure S2: GC chromatographs of the fatty acid profiles of the transgenic lines and wild type of *B. carinata*

Table S1. The 20 most abundant wax ester species of the selected four transgenic lines of *B. carinata*, analyzed by nanoESI-MS/MS

T-line	WE12	WE2		WE3		WE10	
WE-Profile	Mean ± SD (nmol/g seed)	WE-Profile	Mean ± SD (nmol/g seed)	WE-Profile	Mean ± SD (nmol/g seed)	WE-Profile	Mean ± SD (nmol/g seed)
22:1/20:1	4954 ± 2158	22:1/20:1	1930 ± 1600	22:1/20:1	2118 ± 1594	22:1/20:1	1948 ± 1337
22:1/22:1	1975 ± 761	22:1/22:1	698 ± 576	22:1/22:1	590 ± 380	22:1/22:1	598 ± 459
24:1/20:1	836 ± 439	22:1/18:1	382 ± 351	24:1/20:1	325 ± 201	24:1/20:1	300 ± 192
22:1/18:1	793 ± 289	24:1/20:1	278 ± 232	20:1/22:1	249 ± 180	22:1/18:1	260 ± 172
24:1/22:1	593 ± 249	20:1/22:1	207 ± 159	22:1/18:1	240 ± 132	24:1/22:1	173 ± 119
22:2/20:1	531 ± 185	24:1/22:1	199 ± 163	22:0/20:1	188 ± 141	20:1/22:1	171 ± 102
22:1/18:2	455 ± 200	22:1/18:2	160 ± 149	24:0/20:1	183 ± 114	22:2/20:1	154 ± 123
20:1/22:1	446 ± 206	22:2/20:1	145 ± 120	24:1/22:1	179 ± 78	26:1/16:1	127 ± 86
22:1/20:2	362 ± 132	26:1/16:1	125 ± 101	22:2/20:1	159 ± 136	24:0/20:1	125 ± 87
22:1/24:1	320 ± 137	22:0/20:1	121 ± 82	20:0/26:0	156 ± 218	22:1/18:2	124 ± 89
26:1/16:1	309 ± 132	24:1/18:1	118 ± 99	22:1/18:2	136 ± 75	22:0/20:1	111 ± 81
22:2/22:1	307 ± 123	22:1/20:2	116 ± 100	20:1/20:1	133 ± 100	22:1/20:2	108 ± 77
22:0/20:2	298 ± 122	24:0/20:1	114 ± 75	26:1/16:1	125 ± 88	24:1/18:1	103 ± 65
24:0/20:1	295 ± 117	20:1/20:1	112 ± 100	22:1/20:2	123 ± 77	22:0/20:2	103 ± 75
24:1/18:1	284 ± 124	22:0/20:2	112 ± 89	24:1/18:1	105 ± 69	20:1/20:1	97 ± 56
20:0/26:0	282 ± 171	22:1/24:1	104 ± 88	22:1/24:1	87 ± 37	22:1/24:1	94 ± 71
22:0/20:1	278 ± 88	22:1/16:0	104 ± 99	22:0/20:2	86 ± 72	28:1/16:1	83 ± 63
28:1/16:1	251 ± 97	22:2/20:0	94 ± 73	28:1/16:1	84 ± 56	22:2/20:0	79 ± 58
22:0/22:2	247 ± 102	28:1/16:1	92 ± 73	20:1/16:0	76 ± 21	22:1/16:0	71 ± 56
22:2/20:0	244 ± 102	22:0/22:2	84 ± 68	22:2/20:0	75 ± 60	22:2/22:1	69 ± 61
